# Long-Term Consumption of Cuban Policosanol Lowers Central and Brachial Blood Pressure and Improves Lipid Profile With Enhancement of Lipoprotein Properties in Healthy Korean Participants

**DOI:** 10.3389/fphys.2018.00412

**Published:** 2018-04-24

**Authors:** Suk-Jeong Kim, Dhananjay Yadav, Hye-Jeong Park, Jae-Ryong Kim, Kyung-Hyun Cho

**Affiliations:** ^1^Department of Medical Biotechnology, Yeungnam University, Gyeongsan, South Korea; ^2^Research Institute of Protein Sensor, Yeungnam University, Gyeongsan, South Korea; ^3^LipoLab, Yeungnam University, Gyeongsan, South Korea; ^4^Department of Biochemistry and Molecular Biology, Smart-Aging Convergence Research Center, College of Medicine, Yeungnam University, Daegu, South Korea

**Keywords:** policosanol, blood pressure, lipoproteins, apolipoprotein A-I, glycation

## Abstract

Metabolic syndrome is closely associated with higher risk of hypertension, cardiovascular disease (CVD), diabetes and stroke. The aim of the present study was to investigate the long-term effects of policosanol supplementation on blood pressure (BP) and the lipid profile in healthy Korean participants with pre-hypertension (systolic 120–139 mmHg, diastolic 85–89 mmHg). This randomized, double-blinded, and placebo-controlled trial included 84 healthy participants who were randomly assigned to three groups receiving 10 mg of policosanol, 20 mg of policosanol, or placebo for 24 weeks. The BP, lipid profile, and anthropometric factors were measured pre- and post-intervention and then compared. Based on an average of three measurements of brachial BP, the policosanol 20 mg group showed the most significant reduction in average systolic BP (SBP) from 138 ± 12 mmHg at week 0 to 126 ± 13 mmHg at week 24 (*p* < 0.0001). The policosanol 20 mg group also showed significant reductions in aortic SBP and DBP up to 9% (*p* = 0.00057) and 8% (*p* = 0.004), respectively compared with week 0. Additionally, blood renin and aldosterone levels were significantly reduced in the policosanol 20 mg group up to 63% (*p* < 0.01) and 42% (*p* < 0.05), respectively, at week 24. For the blood lipid profile, the policosanol 10 mg and 20 mg groups showed significant reductions in total cholesterol (TC) of around 8% (*p* = 0.029) and 13% (*p* = 0.0004), respectively, at week 24 compared with week 0. Serum HDL-C level significantly increased up to 16% and 12% in the policosanol 10 mg (*p* = 0.002) and 20 mg (*p* = 0.035) group, respectively. The study results suggest that long-term policosanol consumption simultaneously reduces peripheral BP as well as aortic BP accompanied by elevation of HDL-C and % HDL-C in TC in a dose-dependent manner.

## Introduction

Hypertension is a major risk factor for the development of cardiovascular disease (CVD), which is often accompanied by other risk factors such as dyslipidemia (Nelson, 2013; Lee et al., 2017). It is well-known that serum high-density lipoprotein (HDL-C) is inversely correlated with incidence of aging-related diseases such as CVD, diabetes, and Alzheimer's disease (McGrowder et al., 2010; Reitz et al., 2010; Hirano et al., 2014). The HDL-C/total cholesterol (TC) ratio (%), rather than HDL-C (mg/dL), is more important in predicting the risk of incident hypertension (Halperin et al., 2006).

In addition to HDL-C quantity, it has been firmly established that HDL quality and functionality are more important in the suppression of aging-related diseases (Eren et al., 2012). There are a limited number of approaches to increasing HDL-C quantity comprising dietary foods or drugs to elevate the concentration of HDL-C and enhance HDL functionality (Sahebkar et al., 2016). Cuban policosanol (PCO) was reported to lower TC and low-density lipoprotein cholesterol (LDL-C) as well as increase HDL-C levels by inhibiting cholesterol synthesis and increasing LDL-C excretion (Janikula, 2002; Lee et al., 2016; Kim et al., 2017).

Policosanol potentiates the beneficial functions of HDL as well as its antioxidant, anti-glycation, and anti-atherosclerotic activities (Lee et al., 2016; Kim et al., 2017). Although short-term consumption of policosanol has been shown to reduce brachial blood pressure (BP) accompanied by increased HDL-C concentration and antioxidative functionality, correlations of BP reduction with enhancement of HDL-C in TC remain to be investigated in a long-term study.

Quality of HDL is also recognized as important factor in the suppression of atherosclerotic progression. Impaired functionality of HDL such as cholesterol efflux is closely associated with atherosclerotic CVD (Rohatgi et al., 2014).

A previous study reported that short-term consumption of policosanol enhanced HDL-C particle size, and reconstituted HDL (rHDL) containing policosanol increased cholesterol efflux capacity via up-regulation of ABCA1 for excretion (Kim et al., 2017). Although there are inconsistent data related to the efficacy of lowering cholesterol with policosanol therapy (Cubeddu et al., 2006; Francini-Pesenti et al., 2008), Gong et al. (2018) in a recent meta-analysis comprising 22 studies reported that policosanol could be used to lower lipid content and as a safe drug to elevate HDL-C levels (Gong et al., 2018). However, until now, there has been no *in vivo* or human study on the HDL-C/TC ratio and HDL functionality in relation to the central BP-lowering effect of policosanol in human participants over the long-term. In recent years, the prevalence of pre-hypertension is widely increased around the world including Korea (Kim and Lee, 2015). Pre-hypertensive subjects are more risky for causing hypertension and related morbidity. Therefore it is prudent to study the dietary foods or drugs on the pre-hypertensive population to further reduce the actual burden of hypertension and hypertension related mortality in the future. We hypothesized that consumption of policosanol for 24 weeks would reduce both brachial and central aortic BP along with beneficial effects on lipid parameters. Therefore, we tested the physiological effects of policosanol consumption on brachial and central BP, and lipoprotein functionality in healthy Korean subjects.

## Materials and methods

### Policosanol

Policosanol and placebo tablet were obtained from Rainbow & Nature Pty, Ltd (Thornleigh, NSW, Australia). Policosanol (sugar cane wax alcohol, SCWA) consists of considerable chains of alcohol of various lengths. More than 90% of policosanol contents were higher aliphatic alcohols. Individual alcohols present in policosanol are 1-tetracosanol (C_24_H_49_OH; molecular weight—MW: 354.7 mμ) ≤ 2%; 1-hexacosanol (C_26_H_53_OH; MW: 382.4 mμ ≤ 4.5–10%; 1-heptacosanol (C_27_H_55_OH; MW: 396.4 mμ) ≤ 5%; 1-octacosanol (C_28_H_57_OH; MW: 410.5 mμ)) ≤ 60–70%; 1-nonacosanol (C_29_H_59_OH; MW: 424.8 mμ) ≤ 2%; 1-triacontanol (C_30_H_61_OH; MW: 438.5 mμ) ≤ 10–15%; 1-dotriacontanol: (C_32_H_65_OH; MW: 466.5 mμ) ≤ 3–8%; 1-tetratriacontanol (C_34_H_69_OH; MW: 494.5 mμ) ≤ 2%.

### Participants

**Figure 1:**
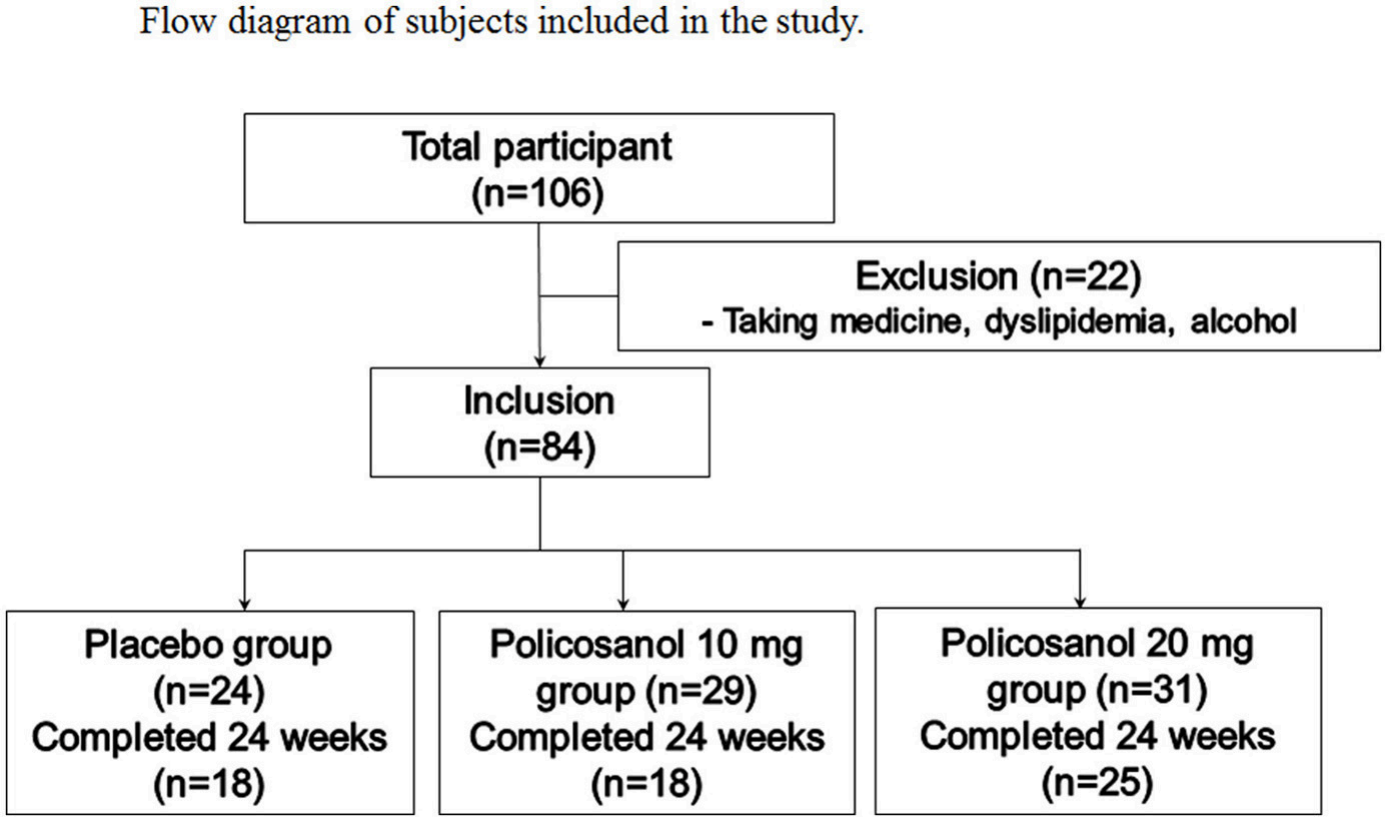
Design of study and subjects. Inclusion criteria were normolipidemic, normoglycemic, and healthy subjects who had prehypertension (systolic 120–139 mmHg, diastolic 80–89 mmHg).

We recruited healthy male and female volunteers who had prehypertension (systolic 120–139 mmHg, diastolic 80–89 mmHg). All recruited participants were pre-screened for eligibility criteria and the inclusion were as follows: age 18–65 years old who had pre-hypertension without any complaint of endocrinological disorder. Heavy alcohol drinkers (>30 g EtOH/day) and those who consumed drugs related to hyperlipidemia, diabetes mellitus, or hypertension were excluded. All participants had unremarkable medical records without illicit drug use or past history of chronic diseases. On the first visit day, all participants casted dice for randomized grouping for group 1 (placebo), group 2 (policosanol 10 mg), and group 3 (policosanol 20 mg). The selected participants consumed policosanol for 24 weeks, based on the represented study design (Figure 1). The study was approved by the Institutional Review Board at Yeungnam University (Gyeongsan, South Korea) endorsed the protocol (IRB #7002016-A-2016-021) and the participants signed an informed consent form prior to research commencement.

### Study design

This study was a double-blinded, randomized, and placebo-controlled trial with a 24-week treatment periods. Participants were directed to take one tablet per day containing policosanol either 10 or 20 mg of sugar cane wax alcohol or placebo consisting of a dextrin and lactose mixture, manufactured by CosmaxBio Inc. (Jecheon, Korea). Other materials used to make the tablets were corn starch, cellulose, gelatin, stearic acid, etc. The ingredients, manufacturing process, and facility were approved by the Korean FDA.

All participants received advice to avoid excess alcohol drinking (less than 30 g of Et-OH per day). They were also instructed to avoid intense exercise (less than 30 min per day at 60–80% maximum capacity). If subjects had a sedentary lifestyle before commencement, we advocated them to balance their lifestyle during the policosanol consumption period to avoid bias due to excess exercise or other life style habits.

### Anthropometric analysis and physical activity assessments

The participants were measured for different anthropometrical parameters such as height, body weight, body mass index (BMI), subcutaneous fat (kg), and visceral fat mass (kg) individually at the same time of day at 4-week intervals using an X-scan plus II body composition analyzer (Jawon Medical, Gyeongsan, Korea).

### Measurement of blood pressure

BP was measured each visit for a total of three times, and the average was recorded at 4-week intervals using three BP measuring instruments. We used a digital BP device (Omron HBP-9020, Kyoto, Japan), mercury sphygmomanometer, and the SphygmoCor system (AtCor Medical, Sydney, Australia) to measure peripheral and central aortic BP (Yadav et al., 2018). All manual measurements or procedures were carried out by a licensed technician (S.J.K.).

The idea of using three different techniques was to obtain more accurate results, as a previous report suggested discrepancies in BP measurement between these devices (Li et al., 2013). Instruments such as the Omron digital BP device and mercury sphygmomanometer are commonly used in hospitals to estimate BP. However, in epidemiological studies, focusing on central aortic BP is limited, and therefore we represent all data in this long-term policosanol study. It is known that central aortic BP provides better prognostic information than brachial BP due to close proximity to important organs such as the heart, brain and kidneys. A meta-analysis of 11 longitudinal studies reported that measurement of central aortic BP could have a good clinical significance and be a better predictor of cardiovascular events (Vlachopoulos et al., 2010). Central aortic pressure waveform represents different components such as augmentation pressure and augmentation index. Augmentation pressure is the difference between early aortic systolic pressure and late aortic systolic pressure. Augmentation index is measured based on the ratio of augmentation pressure to central aortic pulse pressure.

### Blood analysis

Blood was obtained from participants after overnight fasting. Blood was collected using a vacutainer (BD Biosciences, Franklin Lakes, NJ, USA) containing EDTA (final concentration of 1 mM) at weeks 0 and 24 by low-speed centrifugation (3,000 g) and stored at −80°C until analysis. To analyze plasma, total cholesterol (TC), triglyceride (TG), high-density lipoprotein cholesterol (HDL-C), and glucose levels were measured using commercially available kits (Cleantech TS-S; Wako Pure Chemical, Osaka, Japan).

### Angiotensin-converting enzyme and aldosterone-renin analysis

Plasma aldosterone and renin level were measured by radioimmuno assay (RIA) using an instrument (1470-Gamma Counter, PerkinElmer) via the Seegene Medical Foundation (Seoul, Korea). Angiotensin-converting enzyme (ACE) activity was measured by an enzymatic assay kit obtained from Buhlmann Laboratories (Schönenbuch, Switzerland). Homocysteine was measured by chemiluminescent immunoassay using a Liquid stable Reagnet kit obtained from Axis-Shield Diagnostics Ltd. (Luna Place Technology Park, United Kingdom) via the Seegene Medical Foundation (Seoul, Korea).

### Characterization of lipoproteins

Very low-density lipoprotein (VLDL, d < 1.019 g/mL), low-density lipoprotein (LDL, 1.019 < d < 1.063), high-density lipoprotein_2_ (HDL_2_, 1.063 < d < 1.125), and high-density lipoprotein_3_ (HDL_3_, 1.125 < d < 1.225) were isolated from individual plasma samples of each group via sequential ultracentrifugation (Havel et al., 1955), and density was adjusted by addition of NaCl and NaBr in accordance with standard protocols. Samples were centrifuged for 22 h at 10°C and 100,000 g using a Himac CP-100NX (Hitachi, Tokyo, Japan) at the Instrumental Analysis Center of Yeungnam University. To analyze lipoproteins, TC and TG levels were measured using commercially available kits (Cleantech TS-S; Wako Pure Chemical, Osaka, Japan). Protein concentrations of lipoproteins were determined via Lowry protein assay, as modified by Markwell et al. (1978). Expression levels of apoA-I (28 kDa) were determined by SDS-PAGE and immunodetection using apoA-I polyclonal antibody (ab7613) obtained from Abcam (Cambridge, UK).

To assess the degree of lipoprotein oxidation, the concentration of oxidized species in lipoproteins was determined by the thiobarbituric acid reactive substance (TBARS) assay method using malondialdehyde as a standard (Blois, 1958). Extent of glycation between the three groups based on advanced glycation end products (AGEs) in HDL_2_ and HDL_3_ lipoproteins was determined from fluorometric intensities at 370 nm and 440 nm as described previously (McPherson et al., 1988), using a spectrofluorometer LS55 (Perkin Elmer, Shelton, CT, USA) with the WinLab software package (version 4.0).

### LDL oxidation

LDL was oxidized by incubation of the LDL fraction with CuSO_4_ (final concentration of 10 μM) for 4 h at 37°C. oxLDL was then filtered through a 0.22-μm filter (Millex; Millipore, Bedford, MA) and analyzed by thiobarbituric acid reactive substances (TBARS) assay to determine the extent of oxidation using a malondialdehyde (MDA) standard (Blois, 1958). To verify spectroscopic data, oxidized samples were subjected to 0.5% agarose gel electrophoresis to compare electromobilities. The migration of each lipoprotein was dependent on its intact charge and size. Gels were then dried and the bands stained with 0.125% Coomassie Brilliant Blue.

### Data analysis

All data are expressed as the mean ± SD from at least three independent experiments with duplicate samples. For human data analysis, all data were analyzed by normality test; normally distributed data were compared using Student *t*-test. Correlation analysis was carried out using Pearson's test. *p* < 0.05 was defined as being significant. Statistical analysis was performed using the SPSS software program (version 23.0; SPSS, Inc., Chicago, IL, USA).

## Results

### Comparison of body composition, brachial BP, and aortic BP after 24 weeks of therapy

Table 1 represents the data on body composition, peripheral BP, and central BP at baseline (week 0) and after 24 weeks. After 24 weeks of policosanol consumption, there were no significant changes in BMI, as measured values among all groups were around 22–24 (kg/m^2^). Subcutaneous fat and visceral fat contents were similar among all groups between weeks 0 and 24.

**Table 1:** Change of blood pressure profile after 24 weeks consumption.

		**Group 1 Placebo (*n* = 24, M14/F10)**		**Group 2 Policosanol 10 mg (*n* = 29, M21/F8)**		**Group 3 Policosanol 20 mg (*n* = 31, M22/F9)**	
Age		32 ± 14		34 ± 16		31 ± 12	
**Body composition**		**Week 0**	**Week 24**	**Week 0**	**Week 24**	**Week 0**	**Week 24**
BMI		22.3 ± 1.5	22.5 ± 1.4	23.2 ± 1.8	23.4 ± 1.8	23.7 ± 1.5	23.8 ± 1.3
Subcutaneous fat (kg)		12.3 ± 2.3	12.4 ± 2.1	13.6 ± 2.5	14.1 ± 2.7	13.8 ± 2.5	14.4 ± 1.8
Visceral fat (kg)		1.7 ± 0.5	1.6 ± 0.4	2.0 ± 0.5	2.1 ± 0.6	2.0 ± 0.5	2.1 ± 0.4
**Peripheral BP (mmHg)**		**Week 0**	**Week 24**	**Week 0**	**Week 24**	**Week 0**	**Week 24**
SphygmoCor	Systolic	135 ± 12	132 ± 11	139 ± 16	130 ± 15^**^	136 ± 8	123 ± 11^***^
	Diastolic	88 ± 8	84 ± 8	89 ± 10	87 ± 11	87 ± 7	81 ± 9^*^
Omron	Systolic	134 ± 12	135 ± 10	140 ± 18	134 ± 20^*^	138 ± 11	122 ± 10^***^
	Diastolic	84 ± 9	83 ± 9	85 ± 12	84 ± 15	82 ± 10	72 ± 10^**^
Mercury	Systolic	131 ± 6	129 ± 8	134 ± 12	130 ± 17^*^	135 ± 6	121 ± 9^***^
	Diastolic	83 ± 5	81 ± 8	84 ± 10	82 ± 10	84 ± 5	75 ± 9^*^
Average	Systolic	138 ± 9	132 ± 6	135 ± 13	128 ± 6^**^	138 ± 12	126 ± 13^***^
	Diastolic	87 ± 6	83 ± 6	86 ± 10	83 ± 12	87 ± 7	79 ± 8^**^
**Aortic BP (mmHg)**		**Week 0**	**Week 24**	**Week 0**	**Week 24**	**Week 0**	**Week 24**
Aortic	Systolic	123 ± 12	117 ± 9	130 ± 14	120 ± 13^*^	123 ± 13	113 ± 12^***^
	Diastolic	90 ± 8	85 ± 7	92 ± 10	86 ± 8^*^	91 ± 7	84 ± 5^**^
Mean arterial pressure		104 ± 9	100 ± 8	104 ± 11	97 ± 11^*^	103 ± 7	93 ± 9^**^
Pulse pressure		32 ± 8	32 ± 4	35 ± 10	30 ± 5	32 ± 5	29 ± 5

*Data are expressed as mean±SD*.

*** p < 0.001 vs. week 0;

** p < 0.01 vs. week 0;

* *p < 0.05 vs. week 0 in each group. AU, arbitrary unit; BP, blood pressure; BMI, body mass index*.

From the SphygmoCor measurements, there was no significant reduction of BP in group 1, whereas, groups 2 and 3 showed significant SBP reductions up to 7% (130 ± 15 mmHg, *p* = 0.0335) and 10% (123 ± 11 mmHg, *p* = 0.0003), respectively. Especially in group 3, brachial SBP and DBP were reduced to normal level around 126 and 79 mmHg, respectively) after 24 weeks of policosanol therapy. Omron digital BP monitor measurement also revealed that group 3 showed normal SBP and DBP with 12% (134 ± 20 mmHg, *p* = 0.001) and 13% reductions (122 ± 10 mmHg, *p* = 0.004), respectively, compared with week 0. Mercury sphygmomanometer measurement revealed that group 3 showed normal range of SBP and DBP at week 24 with 11% reduction (121 ± 9 and 75 ± 9 mmHg, *p* = 0.001) compared with week 0. Similar to the above, groups 1 and 2 did not show any significant change in SBP or DBP.

Based on an average of three measurements, at week 24, group 3 (policosanol 20 mg) showed significantly reduced average SBP and DBP levels up to 10% (126 ± 13 mmHg, *p* < 0.0001) and 9% (79 ± 8 mmHg, *p* < 0.0001), respectively, whereas group 2 showed 6% reductions in SBP (128 ± 6 mmHg, *p* = 0.016) and DBP (83 ± 12 mmHg, *p* = 0.014) compared with week 0.

Central BP measurements showed that aortic SBP and DBP in group 3 were reduced up to 10% (113 ± 12 mmHg, *p* = 0.001) and 8% (84 ± 5 mmHg, *p* = 0.004) at 24 weeks, compared with week 0. Group 2 showed an 8% reduction in SBP (120 ± 13 mmHg, *p* = 0.001) and 7% reduction in DBP (86 ± 8 mmHg, *p* = 0.014) after 24 weeks, whereas group 1 did not any show significant change. Mean arterial pressure at week 24 was reduced in groups 2 and 3 up to 7 mmHg (97 ± 11 mmHg, *p* < 0.05) and 10 mmHg (93 ± 9 mmHg, *p* < 0.01) respectively, whereas group 1 showed no change. These results suggest that policosanol consumption for 24 weeks significantly reduced aortic BP as well as brachial (peripheral) BP in a dose-dependent manner.

### Changes in serum lipid profile after 24 weeks of therapy

Table 2 indicates the changes of blood profile after 24 weeks of therapy. Regarding blood TC level, groups 2 and 3 showed significant reductions in TC of around 8% (180 ± 8 mg/dL, *p* = 0.029) and 10% (161 ± 7 mg/dL, *p* < 0.001), respectively, whereas blood TG level did not change significantly. Serum HDL-C level did not change in group 1 (around 39 mg/dL) over 24 weeks, whereas group 2 showed a significant increase from 36 ± 2 to 42 ± 3 mg/dL. Group 3 showed the most significant increase in HDL-C level from 39 ± 2 mg/dL at week 0 to 44 ± 2 mg/dL (*p* = 0.007). Group 1 did not show any change in the HDL-C/TC ratio (% HDL-C) between week 0 and 24, and the calculated value was around 22%. Group 3 showed an HDL/TC ratio of 28% (*p* = 0.00014) at week 24 compared to 21% at week 0. Group 3 showed the largest reduction in calculated LDL-C up to 20% (101 ± 30 mg/dL), whereas groups 2 showed 14% reductions, respectively. Blood glucose level was not altered in group 1, whereas groups 2 and 3 showed significant reductions up to 8% (90 ± 11 mg/dL, *p* = 0.03 and 86 ± 12 mg/dL, *p* = 0.05, respectively). Homocysteine level was also similar between the groups at weeks 0 and 24.

**Table 2:** Change of blood profile after 24 weeks policosanol consumption.

	**Group 1 Placebo (*n* = 24, M14/F10)**		**Group 2 Policosanol 10 mg (*n* = 29, M21/F8)**		**Group 3 Policosanol 20 mg (*n* = 31, M22/F9)**	
**Blood profile**	**Week 0**	**Week 24**	**Week 0**	**Week 24**	**Week 0**	**Week 24**
TC (mg/dL)	197 ± 10	184 ± 8	195 ± 7	180 ± 8^*^	185 ± 10	161 ± 7^***^
TG (mg/dL)	81 ± 7	91 ± 7	96 ± 13	97 ± 14	104 ± 17	97 ± 8
HDL-C (mg/dL)	39 ± 2	39 ± 3	36 ± 2	42 ± 3^**^	39 ± 2	44 ± 2^*^
% HDL-C in TC	21.4 ± 1.5	22.3 ± 1.3	18.8 ± 1.1	24.4 ± 2^**^	21.8 ± 1.9	28.0 ± 1.3^***^
TG/HDL-C	2.2 ± 0.2	2.1 ± 0.2	2.8 ± 0.5	2.5 ± 0.4	2.6 ± 0.6	2.2 ± 0.2
LDL-C (mg/dL)	132 ± 35	123 ± 27	131 ± 29	113 ± 25	126 ± 40	101 ± 30^*^
Glucose(mg/dL)	87 ± 13	85 ± 11	96 ± 31	90 ± 11^*^	92 ± 16	86 ± 12^*^
Renin (ng/mL/hr)	4.5 ± 1.4	3.9 ± 0.5	4.8 ± 1.1	3.1 ± 0.6^*^	6.5 ± 1.5	2.4 ± 0.4^**^
Aldosterone (ng/dL)	19.5 ± 1.2	19.0 ± 1.6	29.5 ± 2.3	19.6 ± 2.1^*^	24 ± 2.4	14 ± 1.5^*^
Angiotensin converting enzyme (unit)	33 ± 2	34 ± 2	31 ± 3	33 ± 4	34 ± 3	30 ± 3
Homocysteine (μmol/L)	14 ± 3	13 ± 3	14 ± 3	9 ± 1	13 ± 4	10 ± 2

HDL-C, high-density lipoprotein cholesterol; LDL-C, low-density lipoprotein cholesterol; TC, total cholesterol; TG, triglyceride; LDL-C was calculated using Friedwald formula: LDL-C = TC-(HDL-C+TG/5)

* p < 0.05;

** p < 0.01;

*** *p < 0.001 versus week 0 in each group*.

### Renin, aldosterone, and ACE

At week 0, group 3 (Table 2) showed the highest renin level while groups 1 and 2 showed similar renin levels. After 24 weeks, blood renin level was significantly reduced in groups 2 and 3 up to 35% and 63%, respectively, whereas group 1 did not show any significant change. Aldosterone levels in all groups were 19–29 ng/dL at week 0, whereas, groups 2 and 3 showed significant reductions up to 35% (19.6 ± 2.1 ng/dL) and 42% (14 ± 1.5 ng/dL) respectively, compared with week 0. ACE activity was unchanged after 24 weeks, and all groups showed similar level of around 30–34 units at weeks 0 and 24.

### Correlation analysis between brachial blood pressure and lipid profiles

We studied correlations between brachial BP and lipid profiles based on Pearson's correlation coefficient. At baseline, we observed correlations among SBP, DBP, and lipid levels based on total cholesterol (TC), triglyceride (TG), high-density lipoprotein cholesterol (HDL-C), and percentage (%) of HDL-C and TG/HDL-C. Analysis at baseline in the first group revealed that SBP had a positive correlation with DBP and a negative correlation with TG (Table 3). A significant inverse correlation was observed between TC and % of HDL-C. After 24 weeks of therapy in group 1, only SBP was significantly correlated with DBP (*p* = 0.032). The study did not find any significance between BP and lipid profiles after 24 weeks of placebo therapy (Table 3).

**Table 3:** Pearson's correlation analysis at the baseline and after 24 weeks in the placebo group (Group 1).

	**At 0 week**								**At 24 weeks**						
	**SBP**	**DBP**	**TC**	**TG**	**HDL-C**	**%HDL-C**	**TG/HDL-C**		**SBP**	**DBP**	**TC**	**TG**	**HDL-C**	**%HDL-C**	**TG/HDL-C**
SBP	1	0.798^**^	0.127	−0.630^*^	−0.096	−0.189	−0.427	SBP	1	0.574^*^	0.429	0.156	0.180	−0.146	−0.063
DBP		1	0.317	−0.415	−0.338	−0.514	−0.206	DBP		1	0.219	0.008	0.033	−0.155	−0.075
TC			1	−0.207	0.061	−0.639^*^	−0.241	TC			1	0.488	0.124	−0.503	0.322
TG				1	−0.518	−0.212	0.942^**^	TG				1	0.057	−0.292	0.754^**^
HDL-C					1	0.698^**^	−0.761^**^	HDL-C					1	0.786^**^	−0.584^*^
%HDL-C						1	−0.368	%HDL-C						1	−0.733^**^
TG/HDL-C							1	TG/HDL-C							1

*SBP, systolic blood pressure (mmHg); DBP, diastolic blood pressure (mmHg); TC, total cholesterol; TG, triglyceride (mg/dl); HDL-C, high density lipoprotein cholesterol (mg/dl); % HDL, percentage of high density lipoprotein cholesterol; TG/HDL-C, triglyceride ratio high density lipoprotein cholesterol*.

** *Correlation is significant at the 0.01 level (2-tailed)*.

* *Correlation is significant at the 0.05 level (2-tailed)*.

In group 2, SBP was significantly correlated with DBP (*p* = 0.0001) at week 0. None of the other parameters showed statistical significance. However, DBP showed positive correlations with TG and TG/HDL-C levels (Table 4). In group 2, correlations among SBP, DBP, and lipid parameters were mildly improved at week 24. SBP was significantly correlated with measurement of DBP (*r* = 0.555, *p* = 0.039), TG level (*r* = 0.577, *p* = 0.031), and the TG/HDL-C (*r* = 0.700, *p* = 0.005) ratio (Table 4). Additionally, SBP was negatively correlated with HDL-C (*r* = −0.615, *p* = 0.019) and % of HDL-C (*r* = −0.737, *p* = 0.003). The study did not observe any significant values when correlating DBP with lipid parameters.

**Table 4:** Pearson's correlation analysis at the baseline and after 24 weeks of consuming policosanol (10 mg) (Group 2).

	**At 0 weeks**								**At 24 weeks**						
	**SBP**	**DBP**	**TC**	**TG**	**HDL-C**	**%HDL-C**	**TG/HDL-C**		**SBP**	**DBP**	**TC**	**TG**	**HDL-C**	**%HDL-C**	**TG/HDL-C**
SBP	1	0.847^**^	0.132	0.511	−0.192	−0.202	0.478	SBP	1	0.555^*^	0.481	0.577^*^	−0.615^*^	−0.737^**^	0.700^**^
DBP		1	0.398	0.568^*^	−0.370	−0.487	0.553^*^	DBP		1	0.412	0.285	−0.434	−0.493	0.372
TC			1	0.406	−0.031	−0.618^*^	0.338	TC			1	0.266	−0.193	−0.703^**^	0.288
TG				1	−0.511	−0.619^*^	0.985^**^	TG				1	−0.392	−0.478	0.929^**^
HDL-C					1	0.794^**^	−0.627^*^	HDL-C					1	0.822^**^	−0.638^*^
%HDL-C						1	−0.672^**^	%HDL-C						1	−0.666^*^
TG/HDL-C							1	TG/HDL-C							1

*SBP, systolic blood pressure (mmHg); DBP, diastolic blood pressure (mmHg); TC, total cholesterol; TG, triglyceride (mg/dl); HDL-C, high density lipoprotein cholesterol (mg/dl); % HDL, percentage of high density lipoprotein cholesterol; TG/HDL-C, triglyceride ratio high density lipoprotein cholesterol*.

** *Correlation is significant at the 0.01 level (2-tailed)*.

* *Correlation is significant at the 0.05 level (2-tailed)*.

In group 3 at baseline, SBP showed positive correlations, with DBP, TG, and TG/HDL-C and a negative correlation with HDL-C level (Table 5). DBP showed significant positive correlations with TG and TG/HDL-C.

**Table 5:** Pearson's correlation analysis at the baseline and after 24 weeks of consuming policosanol (20 mg) (Group 3).

	**At 0 weeks**								**At 24 weeks**						
	**SBP**	**DBP**	**TC**	**TG**	**HDL-C**	**%HDL-C**	**TG/HDL-C**		**SBP**	**DBP**	**TC**	**TG**	**HDL-C**	**%HDL-C**	**TG/HDL-C**
SBP	1	0.825^**^	0.257	0.485^*^	−0.460^*^	−0.433	0.487^*^	SBP	1	0.896^**^	0.402	0.513^*^	−0.235	−0.507^*^	0.589^**^
DBP		1	0.340	0.631^*^	−0.305	−0.387	0.593^**^	DBP		1	0.492^*^	0.497^*^	−0.119	−0.549^*^	0.549^*^
TC			1	0.585^**^	−0.389	−0.800^**^	0.555^*^	TC			1	0.181	−0.103	−0.881^**^	0.217
TG				1	−0.584^**^	−0.671^**^	0.988^**^	TG				1	0.117	−0.087	0.975^**^
HDL-C					1	0.832^**^	−0.670^**^	HDL-C					1	−0.512^*^	−0.097
%HDL-C						1	−0.703^**^	%HDL-C						1	−0.201
TG/HDL-C							1	TG/HDL-C							1

*SBP, systolic blood pressure (mmHg); DBP, diastolic blood pressure (mmHg); TC, total cholesterol; TG, triglyceride (mg/dl); HDL-C, high density lipoprotein cholesterol (mg/dl); % HDL, percentage of high density lipoprotein cholesterol; TG/HDL-C, triglyceride ratio high density lipoprotein cholesterol*.

** *Correlation is significant at the 0.01 level (2-tailed)*.

* *Correlation is significant at the 0.05 level (2-tailed)*.

After 24 weeks of policosanol therapy at a concentration of 20 mg, the correlation between BP and lipid profile in the group 3 was significantly improved compared to group 1 and group 2. SBP in group 3 was significantly related with measurement of DBP (*r* = 0.896, *p* = 0.0001), TG (*r* = 0.513, *p* = 0.025), % of HDL-C (*r* = −0.507, *p* = 0.027), and TG/HDL-C (*r* = 0.589, *p* = 0.008) (Table 5). Moreover, DBP was positively related to TC (*r* = 0.492, *p* = 0.032), TG (*r* = 0.497, *p* = 0.030), and TG/HDL-C (*r* = 0.549, *p* = 0.015). A negative correlation was observed between DBP and % of HDL-C (*r* = −0.0549, *p* = 0.015).

### Correlation of central aortic blood pressure with lipid parameters after 24 weeks of policosanol therapy

In group 1, after 24 weeks of placebo therapy, central SBP was significantly correlated with central DBP. However, none of the lipid parameters (TG, TC, HDL-C, and % of HDL-C) showed a significant correlation with measurement of central SBP as well as central DBP (Table S1). After 24 weeks of therapy with 10 mg of policosanol (group 2), the correlation between central aortic pressure and the lipid profile improved slightly. Central SBP was significantly correlated with central DBP, TC, and % of HDL-C (Table S2). Moreover, central DBP was significantly correlated with TC, HDL-C, and % of HDL-C. HDL-C levels and % of HDL-C were negatively correlated with central DBP. In group 3, significance level in the correlation study was improved in comparison with group 1 and 2 and central SBP was significantly related to DBP. Additionally, central DBP was correlated with TG levels and TG/HDL-C (Table S3). Central DBP was also found to be significantly correlated with the concentration of TG and TG/HDL-C after 24 weeks of policosanol therapy.

### LDL oxidation

**Figure 2:**
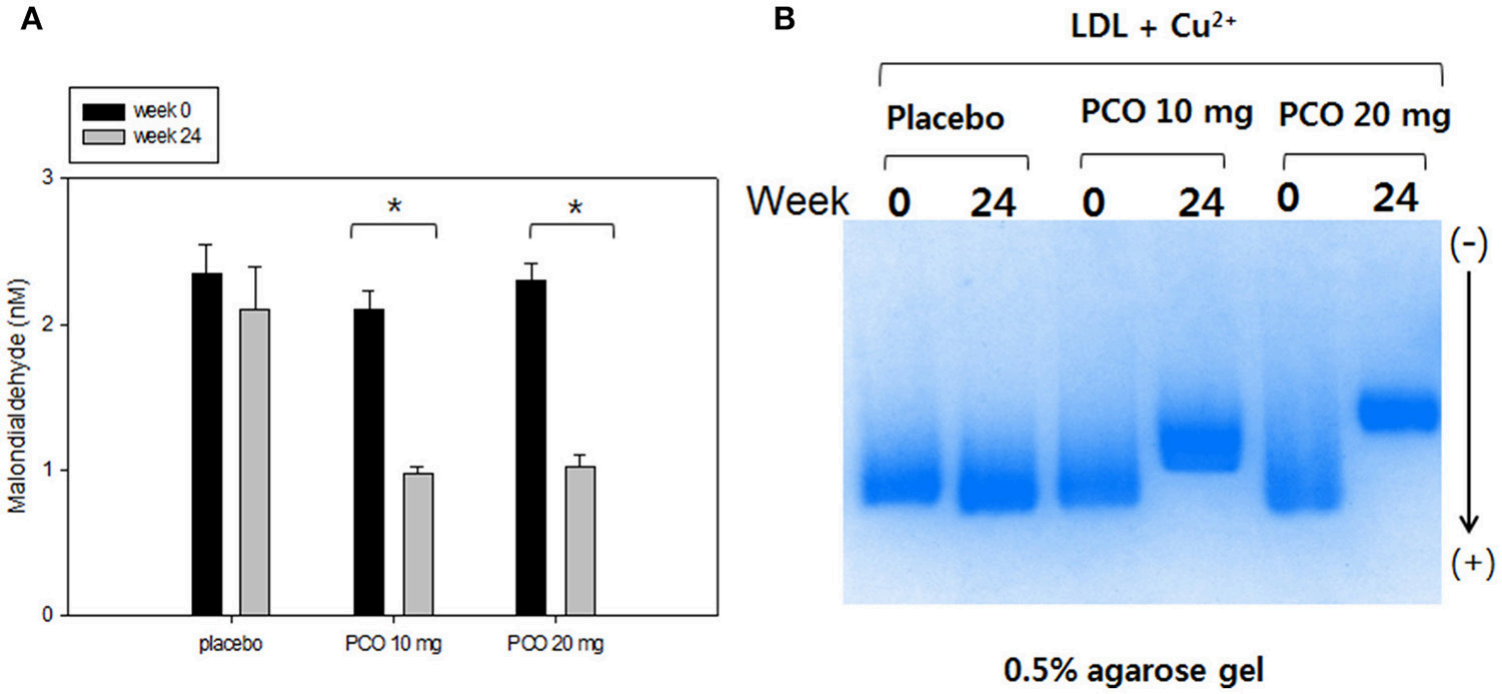
Comparison of LDL oxidation extent before and after policosanol therapy. **(A)** Determination of oxidized species using thiobarbituric acid reactive substances method in LDL (1 mg of protein) in the presence of cupric ion at weeks 0 and 24. ^*^p < 0.05 vs. week 0. **(B)** Comparison of electromobility of LDL between week 0 and 24 with cupric ion in 0.5% agarose gel.

At week 0, all groups showed similar malondialdehyde contents in LDL, as shown in Figure 2A. However, at week 24, groups 2 and 3 showed 57 and 53% reductions in conjugated diene level, whereas group 1 showed 20% reduction. At week 0, LDL from all groups moved to the bottom of the gel with similar electromobilities (Figure 2B). Oxidized LDL moved faster to the cathode position due to an increased negative charge and apo-B fragmentation. At 24 weeks electromobilities of LDL from groups 2 and 3, were much slower compared with week 0, suggesting less production of negatively charged molecules and less fragmentation of apo-B in LDL.

### Glycation extent of lipoproteins

**Figure 3:**
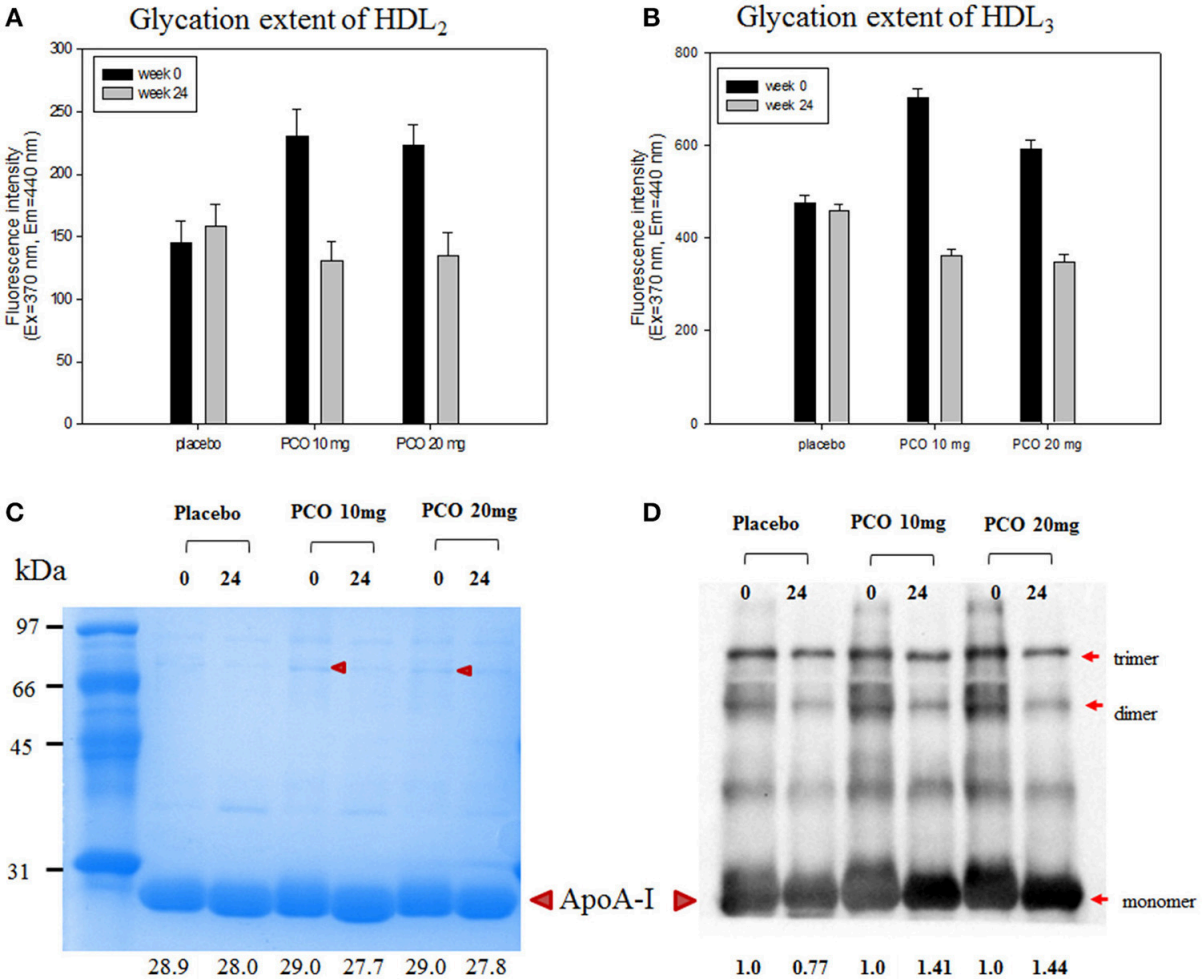
Glycation extent and expression level of apoA-I in HDL_2_ and HDL_3_ at weeks 0 and 24. **(A)** Fluorometric determination (Ex = 370 nm, Em = 440 nm) of glycation extent at weeks 0 and 24 of HDL_2_. **(B)** Fluorometric determination (Ex = 370 nm, Em = 440 nm) of glycation extent at weeks 0 and 24 of HDL_3_. **(C)** Electrophoretic patterns of HDL_3_ from each group at weeks 0 and 24 (5 μg/lane, 15% SDS-PAGE). Lower numbers indicate calculated molecular weight of apoA-I using Chemi-Doc (Bio-Rad). **(D)** Immunodetection of apoA-I in HDL_3_ using anti-human apoA-I antibody (ab7613, Abcam). Lower numbers indicate calculated band intensity of apoA-I using Chemi-Doc (Bio-Rad).

After 24 weeks of policosanol consumption, the policosanol groups showed significantly lowered glycation extent in the HDL_2_ and HDL_3_ fractions, as shown in Figure 3. For the HDL_2_ fraction, groups 2 and 3 showed 44 and 40%, less production of AGEs, respectively, whereas the placebo group showed a similar level of glycation with an 8% increase (Figure 3B). For HDL_3_, groups 2 and 3 showed 48 and 42% less production of AGEs respectively (Figure 3B). These results suggest that glycation extent of HDL was more reduced by policosanol consumption at both 10 and 20 mg.

SDS-PAGE and Western blot analysis for HDL_3_ revealed that the apoA-I band position was slightly lower in after 24 weeks especially in groups 2 and 3, suggesting that electromobility was altered by policosanol consumption due to less glycation (Figure 3C). Immunodetection with apoA-I antibody revealed that apoA-I band intensities in groups 2 and 3 increased by 1.4-fold at week 24, compared with week 0 (Figure 3D). More interestingly, at week 0, groups 2 and 3 showed stronger band intensities for multimerized apoA-I such as dimer and trimer. However, multimeric band intensities were reduced at week 24, suggesting less glycation occurred in the policosanol group.

## Discussion

This study was a randomized clinical trial evaluating the effects of Cuban policosanol on BP and serum lipids in healthy participants who had pre-hypertension. The current results show that 24 weeks of policosanol consumption resulted in significant lowering of brachial (peripheral) BP and central aortic BP in a dose-dependent manner as well as lowering of serum renin and aldosterone levels in pre-hypertensive participants. For the blood lipid parameters, 24 weeks of policosanol therapy resulted in significant reduction of TC and LDL-C as well as concomitant increases in HDL-C and the HDL-C/TC ratio in a dose-dependent manner.

Castaño et al. (2002) reported the effect of policosanol on older patients with hypertension and type II hypercholesterolemia in a prospective, randomized, double-blind, placebo-controlled study. The participants consumed 5–10 mg/day of policosanol for one year, based on the total cholesterol values (6.1 mmol/L). Policosanol treatment group in a long-term significantly lowered the LDL-C (20.5%), TC (15.4%), TG (11.9%), LDL-C/HDL-C ratio (22.2%), and increased HDL-C up to 12.7%. Moreover, policosanol consumption significantly decreased systolic blood pressure when compared with the values of baseline and placebo (Castaño et al., 2002). Additionally, the efficacy and tolerability of policosanol in patients with high global coronary risk were revealed a significant decrease in serum LDL-C, TC, TG, TC/HDL-C, and LDL-C/HDL-C along with elevating the HDL-C. The treatment was well-tolerated; no drug-related clinical or biochemical adverse effects (AEs) were observed (Castano et al., 1999). The exact mechanism of policosanol on the lowering of lipid levels and blood pressure has not been clearly identified. However, some studies have evaluated that the action of policosanol involves various pathways such as activation of AMP-kinase, down—regulation of HMG-CoA reductase, and cholesteryl ester transfer protein inhibition (Mccarty, 2002; Singh et al., 2006; Kim et al., 2017).

Our current results indicate that the brachial BP lowering effect of policosanol is strongly correlated with TC, HDL-C, and the HDL-C/TC ratio in pre-hypertensive patients. More interestingly, the HDL-C/TC ratio (% HDL-C) showed a more negative correlation [correlation coefficient (r) = −0.737 *p* < 0.001] with reduction of SBP than HDL-C (*r* = −0.615, *p* < 0.05) in group 2. The TG/HDL-C ratio (*r* = 0.700, *p* < 0.001) was showed a strong positive correlation with SBP after policosanol therapy for 24 weeks than TG (*r* = 0.577, *p* < 0.05). The same tendency was observed in group 3, as % HDL (HDL-C/TC ratio) was more negatively correlated with SBP (*r* = −0.507, *p* < 0.05) along with DBP (*r* = −0.549, *p* < 0.05) after 24 weeks of therapy.

In placebo and participants consumed 10 mg of policosanol group, the lipid parameters and biomarkers did not vary when comparing values at baseline and after 24 weeks of therapy except for a significant lowering of total cholesterol in group 2 after 24 weeks of 10 mg of policosanol consumption. Participants consumed 20 mg of policosanol clearly showed improvement of the lipid profile (TC, HDL-C, % HDL-C in TC and LDL-C) after consumption of 20 mg of policosanol for 24 weeks. Within the same group, renin and aldosterone levels were reduced at 24 weeks compared to values at baseline. Previous studies reported a positive association of circulatory renin and aldosterone levels with hypertension (Tomaschitz et al., 2010a,b). Moreover, basic and clinical studies strongly suggested an association of the renin angiotensin-aldosterone system (RAAS) with pathogenesis of hypertension (Perticone et al., 2001; Atlas, 2007). Therefore, our study investigated the role of renin and aldosterone hormone in determining the plasma levels of the participants before and after policosanol therapy. Our study reported that consumption of 20 mg of policosanol for 24 weeks resulted in significant lowering of renin and aldosterone levels. There was a slight reduction in the levels of ACE in group 3 after 24 weeks, although the change was not significant. The concentration of homocysteine was not altered in any group after 24 weeks of therapy. This result is corroborated by a previous finding in which participants with metabolic syndrome showed no direct relationship between homocysteine and RAAS (Zacharieva et al., 2008). However, homocysteine levels are independently associated with risk of causing hypertension, as demonstrated from previous studies (Wang et al., 2014; Yang et al., 2017).

In addition to the consideration of brachial and central aortic BP, we measured mean arterial pressure (MAP) in this study which defines the average arterial pressure during a single cardiac cycle and was estimated using the formula (SBP + 2 × DBP)/3. Our study evaluated the MAP, which is more advantageous in diagnosing hypertension and related morbidity. Group 1 did not show any significant difference in MAP after 24 weeks of study. However, after consumption of policosanol at 10 mg and 20 mg per day for 24 weeks, groups 2 and group 3 showed MAP levels of 97 ± 11 mmHg (*p* < 0.05) and 93 ± 9 mmHg (*p* < 0.001) respectively. This result shows that policosanol not only affected central BP, but also helped to alleviate MAP. A higher MAP is correlated with higher incidence of hypertension (Sesso et al., 2000; Yadav et al., 2017) and metabolic syndrome (Hsu et al., 2015). Regarding the importance of aortic BP measurement, while brachial BP is determined by cardiac output and peripheral vascular resistance, central aortic pressure additionally determined by the stiffness of the conduit vessels and the timing/magnitude of pressure wave reflections (Mitchell et al., 2003).

Consumption of policosanol for 24 weeks resulted in significant reduction of central aortic SBP and DBP, indicating that policosanol could lower burden on the left ventricle and risk of CVD. Central aortic pressure is subjected to higher alteration by allopathic drugs than peripheral BP. For management of hypertension, antihypertensive drugs show differential effects on central pressure compared to their effects on brachial pressure (Morgan et al., 2004; Williams et al., 2006; Mackenzie et al., 2009). Dhakam et al. (2006) reported that inhibitors of the RAAS have a significantly greater reduction effect on central pressure compared to brachial pressure (Dhakam et al., 2006). In the REASON trial, normalization of brachial SBP was achieved along with significant greater reduction of central SBP using a low-dose combination of perindopril/indapamide therapy but not atenolol (London et al., 2004). Hypertension treatment based on central BP is more useful than brachial pressures as discussed in recent reports (Sharman et al., 2013; McEniery et al., 2014).

We previously reported that policosanol could inhibit glycation and oxidation *in vitro* (Lee et al., 2016) and *in vivo* (Kim et al., 2017). It is well-known that oxidized LDL is an independent risk factor for CVD and metabolic syndrome (Holvoet et al., 2008). OxLDL is a potent inflammatory trigger of atherosclerosis and vascular complications. Policosanol consumption at both 10 and 20 mg resulted in less production of oxLDL (Figure 2). Few reports on policosanol administration in animals such as in rat and rabbits exhibited a beneficial effects on plaque composition, stability and preventive effects on atherosclerotic lesions including form cell formation (Noa et al., 1995, 1996; Arruzazabala et al., 2000). However, the beneficial effects of policosanol on atherosclerosis development in human remain to be elucidated and future studies are needed to explore whether policosanol reduces the progression of atherosclerosis.

The pathological mechanism of glycation is initiated by non-enzymatic attachment of carbohydrates to blood proteins, especially hemoglobin, and lipoproteins (Nawale et al., 2006). It is well-known that glycation promotes development of aging and metabolic syndrome, including premature atherosclerosis via damage of key proteins. Glycation reduces stability of apoA-I, causing impairment of functionality in type 2 diabetes patients (Kashyap et al., 2017). Policosanol consumption remarkably lowered glycation extent in both HDL_2_ and HDL_3_ (Figures 3A,B) along with less multimerization of apoA-I. Since multimerization of apoA-I is directly associated with aggregation and the amyloidogenic process, dysfunctional HDL is more rapidly produced. Glycation of HDL also triggered more oxidative stress and blood vessel stiffness, resulting in incidence of hypertension. However, policosanol consumption also inhibited multimerization of apoA-I and increased content of content of monomeric apoA-I accompanied by a lower molecular weight band position (Figure 3D). Since glycated apoA-I showed higher molecular weight with smear band intensity as in our previous report (Park et al., 2010), clearer band intensity along with a lower molecular weight band position for apoA-I at week 24 indicates that glycation of HDL was prevented by policosanol consumption.

In conclusion, 24 weeks of policosanol consumption significantly reduced BP accompanied by enhancement of the lipid profile and lipoprotein properties, including reduction of TC, TG/HDL-C, and LDL-C as well as elevation of HDL-C and % HDL-C, resulting in improved anti-oxidant and anti-glycation activities. These results suggest that treatment of hypertension is possible via improvement of the lipid profile and lipoprotein functionality by policosanol therapy.

## Author contributions

S-JK and DY: Performed experiments; H-JP and J-RK: Analyzed data; K-HC: Wrote the manuscript and supervised the whole project.

### Conflict of interest statement

The authors declare that the research was conducted in the absence of any commercial or financial relationships that could be construed as a potential conflict of interest.
