# Supplementary material for: Long-Term Consumption of Cuban Policosanol Lowers Central and Brachial Blood Pressure and Improves Lipid Profile With Enhancement of Lipoprotein Properties in Healthy Korean Participants
Source: Front Physiol. 2018 Apr 24;9:412. doi: 10.3389/fphys.2018.00412 (PMC5939616; doi:10.3389/fphys.2018.00412)
Supplement: Supplementary file 2 [file Table_2.DOCX]

Table S2. Pearson’s correlation analysis between central aortic blood pressure and lipid parameters after 24 weeks of consuming policosanol (10 mg) (group 2).

| Parameters | 24 weeks | | | | | | |
| --- | --- | --- | --- | --- | --- | --- | --- |
|  | CASBP | CADBP | TC | TG | HDL-C | %HDL-C | TG/HDL-C |
| CASBP | 1 | 0.776^**^ | 0.556^*^ | 0.298 | -0.468 | -0.659^*^ | 0.398 |
| CADBP |  | 1 | 0.607^*^ | 0.320 | -0.585^*^ | -0.726^**^ | 0.403 |
| TC |  |  | 1 | 0.266 | -0.193 | -0.703^**^ | 0.288 |
| TG |  |  |  | 1 | -0.392 | -0.478 | 0.929^**^ |
| HDL-C |  |  |  |  | 1 | 0.822^**^ | -0.638^*^ |
| %HDL-C |  |  |  |  |  | 1 | -0.666^**^ |
| TG/HDL-C |  |  |  |  |  |  | 1 |

CASBP, Central aortic systolic blood pressure (mmHg); CADBP, Central aortic diastolic blood pressure (mmHg); TC, total cholesterol; TG, triglyceride (mg/dl); HDL-C, high density lipoprotein cholesterol (mg/dl); % HDL, percentage of high density lipoprotein cholesterol; TG/HDL-C, triglyceride ratio high density lipoprotein cholesterol. **. Correlation is significant at the 0.01 level (2-tailed). *. Correlation is significant at the 0.05 level (2-tailed).
