# Supplementary material for: Long-Term Consumption of Cuban Policosanol Lowers Central and Brachial Blood Pressure and Improves Lipid Profile With Enhancement of Lipoprotein Properties in Healthy Korean Participants
Source: Front Physiol. 2018 Apr 24;9:412. doi: 10.3389/fphys.2018.00412 (PMC5939616; doi:10.3389/fphys.2018.00412)
Supplement: Supplementary file 3 [file Table_3.DOCX]

| Parameters | 24 weeks | | | | | | |
| --- | --- | --- | --- | --- | --- | --- | --- |
|  | CASBP | CADBP | TC | TG | HDL-C | %HDL-C | TG/HDL-C |
| CASBP | 1 | 0.913^**^ | 0.195 | 0.456^*^ | -0.058 | -0.287 | 0.491^*^ |
| CADBP |  | 1 | 0.322 | 0.486^*^ | -0.061 | -0.337 | 0.504^*^ |
| TC |  |  | 1 | 0.212 | -0.008 | -0.850^**^ | 0.232 |
| TG |  |  |  | 1 | 0.181 | -0.076 | 0.972^**^ |
| HDL-C |  |  |  |  | 1 | 0.489^*^ | -0.046 |
| %HDL-C |  |  |  |  |  | 1 | -0.201 |
| TG/HDL-C |  |  |  |  |  |  | 1 |

Table S3. Pearson’s correlation analysis between central aortic blood pressure and lipid parameters after 24 weeks of consuming policosanol (20 mg) (group 3).

CASBP, Central aortic systolic blood pressure (mmHg); CADBP, Central aortic diastolic blood pressure (mmHg); TC, total cholesterol; TG, triglyceride (mg/dl); HDL-C, high density lipoprotein cholesterol (mg/dl); % HDL, percentage of high density lipoprotein cholesterol; TG/HDL-C, triglyceride ratio high density lipoprotein cholesterol. **. Correlation is significant at the 0.01 level (2-tailed). *. Correlation is significant at the 0.05 level (2-tailed).
